# Tissue-Specific Transcriptomics Uncover Exercise-Responsive Immune–Metabolic Regulatory Targets in Obesity

**DOI:** 10.3390/metabo16070472

**Published:** 2026-07-06

**Authors:** Yingfeng Chen, Renqing Zhao, Ji Ma, Weidong Zheng, Jian Gong

**Affiliations:** 1 Department of Physical Education, Yangzhou Polytechnic University, Yangzhou 225009, China; yingfengchen@yzpc.edu.cn (Y.C.);; 2 College of Physical Education, Yangzhou University, Yangzhou 225009, China; 3 College of Physical Education, Shanghai University of Sport, Shanghai 200438, China

**Keywords:** skeletal muscle, adipose tissue, metabolism, obesity, exercise, *CCL2*

## Abstract

**Highlights:**

**What are the main findings?**

- Obesity induces distinct transcriptional abnormalities in adipose tissues and skeletal muscle. As a tissue-specific intervention, exercise alleviates inflammation and remodels immune and metabolic phenotypes in adipose tissues, while only triggering adaptive changes in energy metabolism in skeletal muscle.
- Transcriptomic network analysis combined with cross-species validation confirms that *CCL2* emerged as a conserved candidate potentially involved in exercise-mediated immune–metabolic recovery in white adipose tissue.

**What are the implications of the main findings?**

- Exercise exerts distinct regulatory mechanisms in adipose tissues and skeletal muscle.
- *CCL2* represents a promising candidate for further investigation.

**Abstract:**

**Objectives**: Obesity disrupts adipose and systemic immune–metabolic homeostasis, yet the molecular mechanisms through which exercise restores abnormal tissue function remain incompletely defined. This study aimed to screen cross-tissue candidate genes associated with exercise-mediated correction of obesity-related transcriptional disorders via multi-tissue transcriptome profiling and bioinformatic gene prioritization. **Methods**: Transcriptomic datasets of mouse visceral white adipose, subcutaneous white adipose and skeletal muscle were downloaded from the public GEO database, covering normal control, high-fat induced obese and post-exercise intervention groups. R programming was applied to complete differential analysis, GO/KEGG enrichment, PPI network, LASSO and GSEA; independent human adipose datasets from GEO validated candidate genes. **Results**: Exercise reversed obesity-triggered transcriptional changes in adipose tissues. Exercise-responsive genes concentrated on immune inflammation, lipid and energy metabolism. Key hub genes for tissue remodeling were screened, and depot-specific pathway regulation was verified by GSEA. *CCL2* showed consistent expression trends across mouse and human adipose data. **Conclusions**: This study identifies distinct tissue-specific transcriptional responses to exercise: visceral adipose mainly achieves reversal of obesity-induced inflammatory dysregulation, subcutaneous adipose undergoes combined immune–inflammatory and metabolic reprogramming, while skeletal muscle presents only energy-metabolism adaptive remodeling without obvious reversal of obese gene disorders. Immune–metabolic pathways dominate exercise-induced restoration in adipose tissues. Integrated network screening and cross-species validation identified *CCL2* as a conserved candidate associated with exercise-responsive immune–metabolic pathways, providing valuable molecular candidates for further anti-obesity research.

## 1. Introduction

Obesity is a prevalent chronic metabolic disorder characterized by adipose tissue (AT) hyperplasia and dysfunction, and has become a major global public health concern [1]. Long-term obesity induces lipid accumulation, insulin resistance and chronic low-grade inflammation, increases the risk of various metabolic complications, and impairs healthspan [2]. As an effective non-pharmacological intervention, exercise modulates skeletal muscle function, glucose metabolism and cardiovascular health, and improves metabolic fitness as well as extending healthspan through multi-system effects [3,4,5].

The systemic metabolic benefits of exercise are largely attributed to its ability to mediate inter-organ and intercellular communication [6]. Extracellular vesicles (EVs) serve as crucial carriers for such crosstalk [7]. Exercise elevates circulating EV levels and remodels their cargo, including proteins, lipids and microRNAs (miRNAs). Accumulating evidence has demonstrated that adipocyte-derived miRNAs can be delivered to distal organs such as the liver via EVs to regulate metabolic and inflammatory processes [8,9]. Obesity disrupts EV-mediated inter-tissue communication and subsequently triggers a range of metabolic disorders, whereas exercise can restore this signaling network and alleviate obesity-related pathological changes [10]. To date, the detailed molecular mechanisms by which exercise regulates metabolic remodeling through this communication pathway remain poorly understood.

Exercise drives comprehensive homeostatic remodeling across cells, tissues and organs, and reverses obesity-induced metabolic disorders via sophisticated molecular mechanisms to restore systemic metabolic balance [11,12]. As a primary responsive tissue to exercise, skeletal muscle (SM) also acts as an endocrine organ to initiate systemic metabolic adaptation upon exercise stimulation [13]. AT is the major target of obesity, and previous studies have indicated distinct responses of different adipose depots to exercise [14,15]. Nevertheless, there is a lack of systematic comparative analysis on the exercise-responsive patterns between visceral white adipose tissue (vWAT) and subcutaneous white adipose tissue (scWAT). Their distinct roles in exercise-induced anti-inflammation and metabolic remodeling, as well as the underlying core molecular mechanisms, remain unclear. Additionally, the synergistic regulatory networks among SM, vWAT and scWAT have not been fully elucidated. Accordingly, this study aims to construct a multi-tissue molecular response atlas of exercise intervention in obese states, and systematically reveal the tissue-specific transcriptomic signatures and regulatory mechanisms of the three key metabolic tissues during exercise-mediated metabolic remodeling.

We hypothesize that obesity specifically disturbs immune and metabolic regulatory networks in vWAT, scWAT and SM, leading to tissue-specific transcriptional dysregulation. The restorative effects of exercise also vary distinctly across different metabolic tissues: vWAT primarily alleviates obesity-induced inflammatory damage, scWAT undergoes dual reprogramming of immune inflammation and metabolism, while SM only presents adaptive changes in energy metabolism and fails to reverse obesity-related transcriptional abnormalities. By integrating multi-tissue transcriptomic analysis, network screening and cross-species validation, this study aims to identify conserved core hub genes governing exercise-mediated metabolic remodeling. Among these candidates, *CCL2* is expected to emerge as a cross-species conserved candidate associated with the restoration of immune–metabolic homeostasis upon exercise intervention. The findings provide a transcriptomic framework for understanding tissue-specific exercise responses in obesity and offer candidate targets for future research, pending independent experimental validation.

## 2. Materials and Methods

### 2.1. Data Collection

The transcriptomic dataset GSE183239 was obtained from the Gene Expression Omnibus (GEO) database [16]. This bulk RNA-seq dataset was derived from 6-week-old male C57BL/6JN mice, consisting of three groups: standard chow control (SC, 10% kcal fat), 6-week high-fat diet-induced obese group (SH, 60% kcal fat), and obese mice receiving an additional 3-week exercise intervention (TH). In the original study, exercise was performed as voluntary wheel running with free access to running wheels; running distance was recorded daily, and mice running less than 3 km/day were excluded. Sequencing was performed on vWAT, scWAT, and SM (triceps muscle) from each animal (*n* = 5 per group) [17].

### 2.2. Identification of Differentially Expressed Genes

Differential expression analysis was conducted using the DESeq2 package [18]. Gene annotation was performed by converting Ensembl IDs to official gene symbols via the org.Mm.eg.db annotation package, and count sums for genes corresponding to multiple probes were aggregated by unique gene symbol [19]. Low-expression genes were filtered by retaining only those with non-zero read counts in at least three samples, followed by DESeq2′s internal pre-filtering step to keep genes with a row sum of counts ≥ 10 across all samples. Log_2_ fold changes were shrunk using the apeglm method. Genes with an adjusted *p*-value < 0.05 and |log_2_FC| > 1 were defined as differentially expressed.

Exercise-reversed genes were defined as genes that were significantly dysregulated in obesity (SH vs. SC, adjusted *p* < 0.05 and |log_2_FC| > 1) and showed a statistically significant opposite expression trend following exercise intervention (TH vs. SH, adjusted *p* < 0.05 and |log_2_FC| > 1), with the direction of log_2_ fold change being opposite between the two comparisons. No additional threshold for the extent of restoration (e.g., percentage recovery toward SC levels) was imposed, as the primary objective was to identify genes whose obesity-induced dysregulation was directionally corrected by exercise. Such reversed genes were screened separately in vWAT, scWAT and triceps muscle; genes without obvious reversal characteristics in SM were excluded from the reversed gene pool. Results were visualized using volcano plots, Venn diagrams and grouping heatmaps.

### 2.3. GO and KEGG Enrichment Analysis

Functional enrichment analysis of exercise-reversed genes from vWAT and scWAT was performed using the clusterProfiler package (v4.18.4) in R [20]. GO analysis [21,22] covered biological process (BP), cellular component (CC), and molecular function (MF). KEGG pathway enrichment [23] was performed to dissect functional discrepancies between two adipose depots: vWAT enrichment focused on inflammation and immune chemotaxis pathways, while scWAT covered both inflammatory and lipid metabolic pathways. For SM, independent GO/KEGG enrichment was conducted on its differential genes to annotate muscle structure and energy metabolism-related biological functions. Terms with adjusted *p* < 0.05 were considered significantly enriched.

### 2.4. PPI Network Construction and Hub Gene Identification

Protein–protein interaction (PPI) networks of exercise-reversed genes from vWAT and scWAT were separately constructed using the STRING database [24]. Network topology was analyzed using Cytoscape software (3.10.2) [25]. Hub genes were screened according to node degree; top hub genes of vWAT were mainly immune-associated genes, whereas hub genes of scWAT participated in both inflammation and metabolic regulation. Higher-degree nodes were defined as core regulatory hub genes.

### 2.5. Feature Gene Selection Using LASSO Regression

Least absolute shrinkage and selection operator (LASSO) regression [26] was applied respectively to exercise-reversed gene expression matrices of vWAT, scWAT, and all differential genes of SM to screen tissue-specific exercise-responsive feature genes. For each tissue, the expression matrix consisted of 15 samples (5 per group: SC, SH, and TH). The dependent variable was the binary group assignment (obese vs. exercise-trained obese), and the independent variables were the normalized expression values of the candidate gene sets. The optimal λ value was determined by 10-fold cross-validation using the one-standard-error rule, and genes with non-zero coefficients under the optimal λ were selected as feature genes. Inter-group expression variation was displayed via heatmaps.

### 2.6. Gene Set Enrichment Analysis (GSEA)

Gene set enrichment analysis [27] was performed on all genes ranked by expression differences between TH and SH groups. Hallmark gene sets related to inflammation, immune response and metabolism were selected as reference sets. GSEA was implemented in a tissue-divided manner: vWAT and scWAT were mainly analyzed for inflammatory signaling and lipid metabolic pathway changes; triceps muscle was subjected to independent GSEA focusing on energy metabolism pathways including oxidative phosphorylation and fatty acid metabolism. Normalized enrichment score (NES), FDR and adjusted *p*-value were used to evaluate significance. Pathways with FDR < 0.25 were considered significantly enriched.

### 2.7. Human Dataset Validation

To verify the cross-species conservation and clinical relevance of candidate genes identified in the mouse model, independent human adipose transcriptomic datasets were retrieved from the GEO database as of March 2026. Datasets were selected based on the following criteria: human origin, transcriptomic profiles derived from white adipose tissue (either subcutaneous or visceral), and availability of expression data from obese individuals to assess obesity-associated expression patterns. Among the datasets meeting these criteria at the time of our search, GSE162653 (human scWAT) and GSE294150 (human vWAT) were selected for validating the adipose-specific candidate genes from our mouse analysis.

GSE162653 (human scWAT) was used to verify expression trends of *CCL2*, *MMP12*, *RASL11A*, *ADISSP*, and *AGPAT2*. In the original study [28], this cohort included 84 participants (39 normal-weight individuals with BMI 18.5–25 kg/m^2^ and 45 individuals with obesity with BMI 30–40 kg/m^2^ and waist circumference ≥94 cm for males or ≥80 cm for females; mean age: 31.68 ± 14.79 years in the normal-weight group and 44.55 ± 12.14 years in the obesity group; sex distribution: 10/28 male/female in the normal-weight group and 12/33 in the obesity group). Of these, 10 samples were subjected to transcriptomic profiling and deposited in GEO; however, the sex distribution for these specific 10 sequenced samples was not explicitly indicated in the public repository.

GSE294150 (human vWAT) was adopted to validate *TREM2*, *TYROBP*, *LGALS3*, *S100A8*, *CCL2*, *LEP*, and other candidate genes. This dataset comprises 40 visceral adipose tissue samples from patients with morbid obesity who underwent laparoscopic sleeve gastrectomy; no detailed demographic information (age or sex) was provided in the GEO submission.

For both datasets, expression trends were compared between human and mouse samples solely to assess the conservation of expression directionality in obesity (i.e., consistent up- or downregulation patterns across species), rather than to establish equivalent exercise responses or perform clinical subgroup analyses.

### 2.8. Statistical Analysis

All statistical analyses and bioinformatic computations were performed in R (version 4.5.2; macOS platform). Differential gene expression analysis was conducted using the DESeq2 package. Functional enrichment analyses for GO and KEGG were performed using the clusterProfiler package. LASSO regression was implemented using the glmnet package, and the optimal penalty parameter λ was determined by 10-fold cross-validation. GSEA was performed using the clusterProfiler package. Statistical significance was defined as adjusted *p* < 0.05 for differential expression and functional enrichment, while FDR < 0.25 was used as the threshold for GSEA. All data processing, statistical analyses, and visualizations including volcano plots, Venn diagrams, and heatmaps were performed within the R environment, except for PPI network visualization, which was conducted using Cytoscape software.

## 3. Results

### 3.1. Differential Gene Expression Analysis and Identification of Exercise-Reversed Genes

To investigate transcriptional alterations associated with obesity and exercise intervention, differential expression analyses were performed across three tissues, including vWAT, scWAT and SM, using the GSE183239 transcriptomic dataset. Following quality control, normalization, and gene ID conversion, the processed expression matrix was used for all downstream analyses.

**Figure 1:**
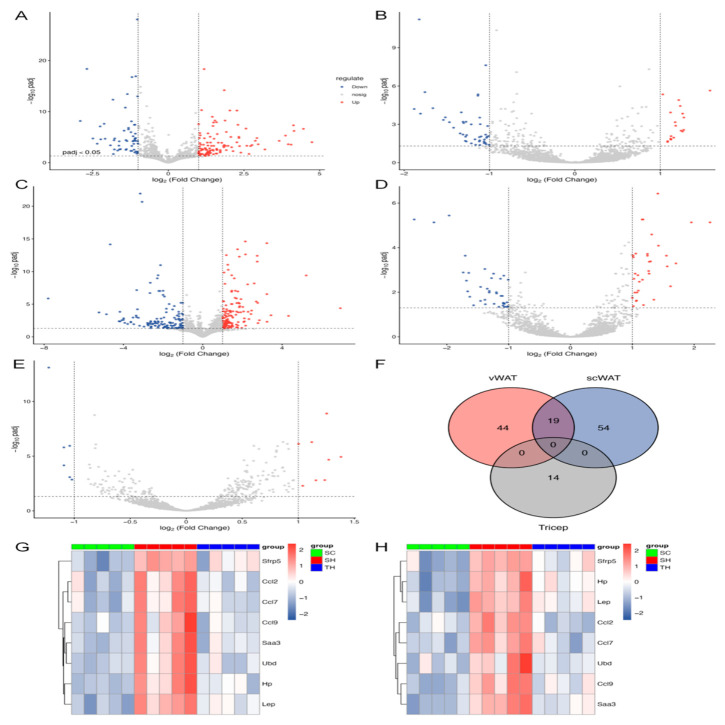
Differential expression analysis and identification of exercise-reversed genes in vWAT, scWAT, and triceps. (**A**–**E**) Volcano plots showing DEGs in each comparison and tissue: (**A**) vWAT (SH vs. SC), (**B**) vWAT (TH vs. SH), (**C**) scWAT (SH vs. SC), (**D**) scWAT (TH vs. SH), (**E**) triceps (TH vs. SH). Red points: significantly upregulated genes; blue points: significantly downregulated genes; gray points: non-significant genes (adjusted *p* < 0.05, |log_2_FC| > 1). (**F**) Venn diagram showing overlapping exercise-reversed genes among vWAT, scWAT, and triceps. (**G**,**H**) Heatmaps of the 8 common exercise-reversed genes in vWAT (**G**) and scWAT (**H**). Rows represent genes (*Ccl2*, *Ccl7*, *Ccl9*, *Hp*, *Lep*, *Saa3*, *Sfrp5*, *Ubd*), and columns represent individual samples. Expression values are normalized by row (z-score), with red indicating high expression and blue indicating low expression. Top color bars denote group assignments: SC (green), SH (red), TH (blue). SC, normal control; SH, sedentary obese; TH, exercise-trained obese.

Using the thresholds of adjusted *p* < 0.05 and |log_2_FC| > 1, obesity-associated differentially expressed genes (DEGs) were identified. In the obesity comparison, 170 DEGs were detected in vWAT, including 113 upregulated and 57 downregulated genes (Figure 1A), whereas 261 DEGs were identified in scWAT, including 136 upregulated and 125 downregulated genes (Figure 1C). In contrast, no significant DEGs were observed in triceps under obese conditions, and therefore, the analysis of exercise-reversed genes was restricted to adipose tissues.

For the exercise intervention comparison (TH vs. SH), 63 DEGs were identified in vWAT (19 upregulated and 44 downregulated; Figure 1B), 73 in scWAT (34 upregulated and 39 downregulated; Figure 1D), and 14 in triceps (eight upregulated and six downregulated; Figure 1E).

To further characterize the regulatory effects of exercise, genes whose obesity-induced dysregulation was reversed following exercise intervention were identified. A total of 45 exercise-reversed genes were detected in vWAT and 26 in scWAT, whereas no evident reversed genes were identified in triceps (Figure 1F). Heatmap analysis showed distinct expression patterns among groups, with these genes exhibiting obesity-associated dysregulation in the SH group and partial restoration toward normal expression levels following exercise intervention in the TH group (Figure 1G,H). Detailed classifications and gene lists of exercise-reversed genes are provided in Table 1.

### 3.2. Functional Enrichment Analysis of Exercise-Reversed Genes in vWAT and scWAT

To characterize the biological functions associated with exercise-reversed genes, GO functional enrichment and KEGG pathway analyses were performed for vWAT and scWAT.

**Figure 2:**
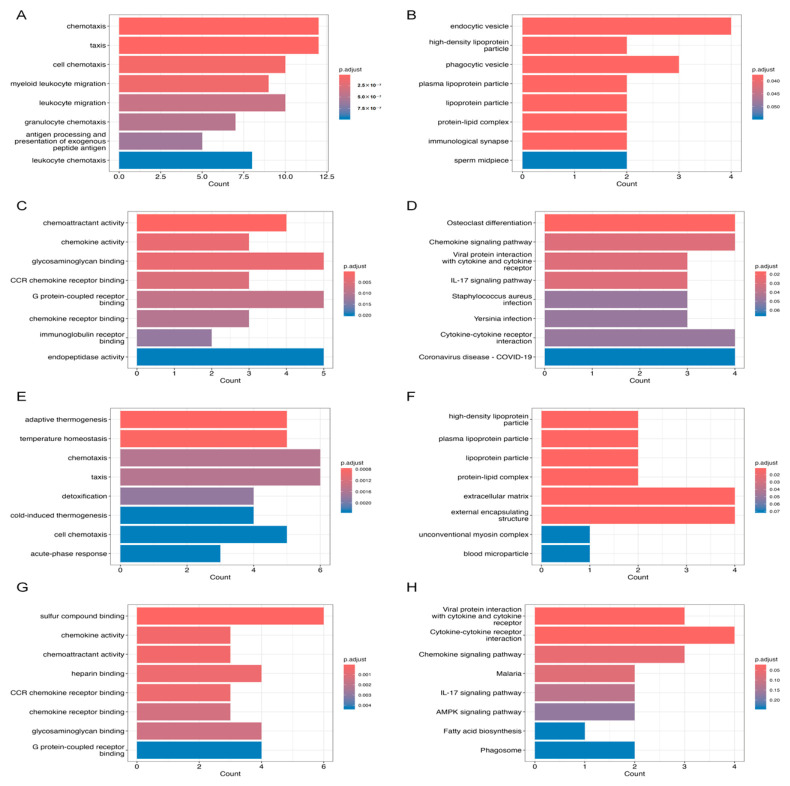
GO and KEGG enrichment of exercise-reversed genes in vWAT (**top**) and scWAT (**bottom**). (**A**–**D**) vWAT: BP, CC, MF and KEGG; (**E**–**H**) scWAT: BP, CC, MF and KEGG. Bar color corresponds to adjusted *p*-value (red = high significance, blue = low significance), bar length indicates enriched gene number.

In vWAT, BP terms were mainly enriched in immune- and inflammation-related processes, including chemotaxis, myeloid leukocyte migration, and leukocyte chemotaxis (Figure 2A). CC terms were primarily associated with the phagocytic vesicle, high-density lipoprotein particle, and immunological synapse (Figure 2B). MF terms were enriched in chemokine activity, CCR chemokine receptor binding, and G protein-coupled receptor binding (Figure 2C). KEGG pathway analysis showed significant enrichment in inflammatory signaling pathways, including the chemokine signaling pathway, IL-17 signaling pathway, and cytokine–cytokine receptor interaction (Figure 2D).

In scWAT, BP terms were enriched in both immune–inflammatory and metabolic processes, including adaptive thermogenesis, temperature homeostasis, and chemotaxis (Figure 2E). CC terms were mainly associated with the extracellular matrix, external encapsulating structure, and high-density lipoprotein particle (Figure 2F). MF terms were enriched in chemokine activity, chemoattractant activity, and heparin binding (Figure 2G). KEGG pathway analysis demonstrated enrichment in both inflammatory and lipid metabolism-related pathways, including the AMPK signaling pathway, fatty acid biosynthesis, and chemokine signaling pathway (Figure 2H).

Comparison of enrichment profiles between the two adipose depots showed that vWAT-reversed genes were predominantly enriched in immune–inflammatory pathways, whereas scWAT-reversed genes were enriched in both inflammatory and lipid metabolism-related pathways, including fatty acid metabolism and cholesterol metabolism.

### 3.3. Protein–Protein Interaction (PPI) Network Construction and Hub Gene Identification of Exercise-Reversed Genes

To identify potential core regulatory genes associated with exercise intervention, PPI networks of exercise-reversed genes in vWAT and scWAT were constructed using the STRING database, and hub genes were identified based on node degree connectivity.

**Figure 3:**
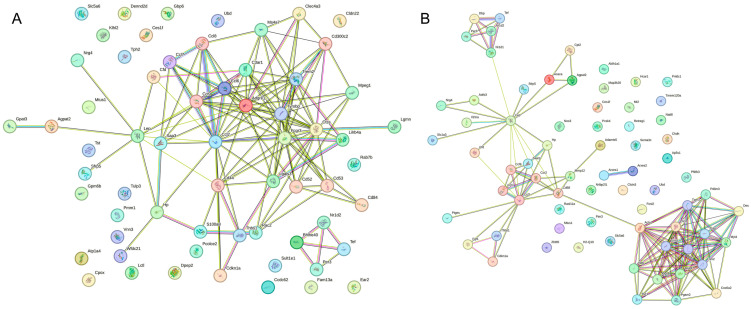
PPI networks of exercise-reversed genes and hub gene identification in vWAT and scWAT. (**A**) PPI network for vWAT reversed genes. A total of 45 genes were included, with key hub genes including *Tyrobp*, *Ctss*, *Adgre1*, *Fcgr3*, and *Ccl2.* Nodes represent proteins, and edges represent interactions between proteins. (**B**) PPI network for scWAT-reversed genes. A total of 26 genes were included, with key hub genes including *Ccl2*, *Lep*, *Cd68*, *Ccl9*, and *Saa3*. Node gradient colors from blue, green, yellow, orange to red/purple correspond to ascending connectivity degree; red and purple nodes represent genes with the highest interaction connectivity. Node diameter positively correlates with the strength of protein interaction.

In vWAT, a total of 45 exercise-reversed genes were included in the PPI network (Figure 3A). The top-ranked hub genes according to degree connectivity were *Tyrobp*, *Ctss*, *Adgre1*, *Fcgr3*, and *Ccl2*. These genes were mainly associated with immune cell activation, chemotaxis, inflammatory signaling, antigen presentation, and phagocytosis.

In scWAT, 26 exercise-reversed genes were included in the PPI network (Figure 3B). The major hub genes included *Ccl2*, *Lep*, *Cd68*, *Ccl9*, and *Saa3*. Compared with vWAT, the hub genes identified in scWAT were associated with both inflammatory responses and metabolic regulation.

The PPI network in vWAT showed a relatively denser immune-related interaction pattern, with hub genes including *Tyrobp*, *Adgre1*, *Trem2*, and *Fcgr3*. In contrast, the scWAT network was centered on *Ccl2* and *Lep* and included genes related to both inflammatory and metabolic processes. These findings demonstrated distinct network characteristics between the two adipose depots.

### 3.4. GSEA of Immune and Metabolic Pathways

To investigate pathway-level alterations associated with exercise intervention in AT, GSEA was performed on hallmark pathways in both vWAT and scWAT.

**Figure 4:**
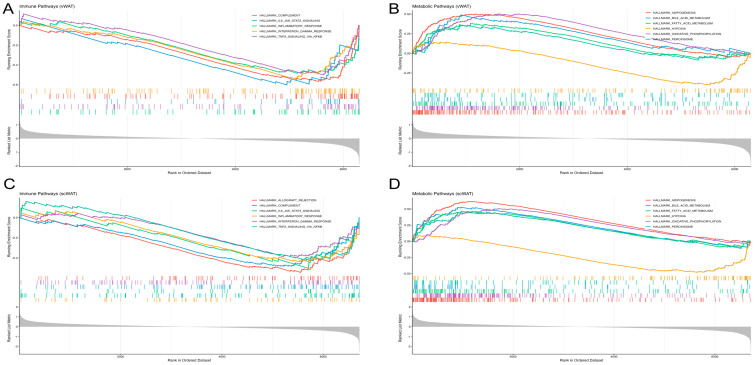
GSEA of hallmark pathways in vWAT and scWAT. (**A**,**B**) Immune–inflammatory pathways in vWAT (**A**) and scWAT (**B**). Top enriched pathways include TNF-α/NF-κB signaling, interferon-γ response, inflammatory response, IL-6/JAK/STAT3 signaling, and complement system. All pathways showed negative enrichment scores (NES < 0, FDR < 0.05), indicating downregulation by exercise. (**C**,**D**) Metabolic pathways in vWAT (**C**) and scWAT (**D**). Key pathways include adipogenesis, fatty acid metabolism, oxidative phosphorylation, peroxisome function, and bile acid metabolism. Metabolic pathways showed stronger enrichment in scWAT, with some pathways presenting positive enrichment scores.

In both vWAT and scWAT, multiple immune–inflammatory pathways were significantly negatively enriched (NES < 0, FDR < 0.05), including the complement system, IL-6/JAK/STAT3 signaling, inflammatory response, interferon-γ response, and TNF-α/NF-κB signaling (Figure 4A,C). Compared with scWAT, vWAT exhibited lower NES and stronger enrichment significance for several inflammatory pathways, particularly interferon-γ response and the complement system.

Metabolic pathway enrichment showed distinct patterns between the two adipose depots (Figure 4B,D). In vWAT, pathways including oxidative phosphorylation, fatty acid metabolism, and peroxisome were negatively enriched (Figure 4B). In scWAT, pathways related to adipogenesis, fatty acid metabolism, and peroxisome exhibited relatively higher enrichment scores (Figure 4D).

Overall, GSEA demonstrated different pathway enrichment profiles between the two ATs. vWAT showed predominant enrichment of immune–inflammatory pathways, whereas scWAT displayed enrichment in both inflammatory and metabolic pathways. These patterns were generally consistent with the results of differential expression analysis, functional enrichment analysis, and PPI network analysis.

### 3.5. LASSO Regression-Based Feature Selection of Key Exercise-Responsive Genes

To further identify representative genes associated with exercise intervention, LASSO regression analysis was performed using exercise-reversed genes for feature selection.

**Figure 5:**
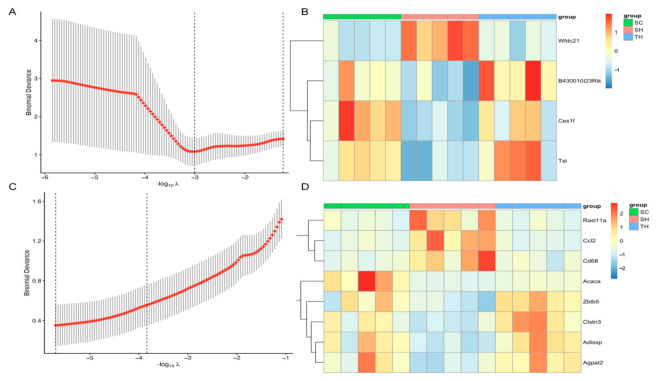
LASSO regression analysis and expression patterns of key exercise-reversed genes in vWAT and scWAT. (**A**,**B**) vWAT results: (**A**) cross-validation curve for LASSO regression, showing binomial deviance as a function of −log_10_(λ). The gray shaded bands denote the standard error of binomial deviance at each λ value; the solid red line represents the mean binomial deviance across cross-validation folds; two vertical dashed lines indicate the λ values for the minimum deviance and one standard error above the minimum deviance, respectively. (**B**) heatmap of the 4 selected vWAT feature genes *(Wfdc21*, *B430010I23Rik*, *Ces1f*, *Tst*) across SC, SH, and TH groups. (**C**,**D**) scWAT results: (**C**) Cross-validation curve for binomial LASSO regression, showing binomial deviance as a function of −log_10_(λ). The gray shaded bands denote the standard error of binomial deviance at each λ value; the solid red line represents the mean binomial deviance across cross-validation folds; two vertical dashed lines indicate the λ values for the minimum deviance and one standard error above the minimum deviance, respectively. (**D**) heatmap of the 8 selected scWAT feature genes (*Rasl11a*, *Ccl2*, *Cd68*, *Acaca*, *Zbtb5*, *Clstn3*, *Adissp*, *Agpat2*) across groups. Heatmap colors represent normalized expression (row z-score; red: high expression, blue: low expression). Group color bars indicate SC (green), SH (red), and TH (blue).

In vWAT, the cross-validation curve showed that binomial deviance gradually decreased and stabilized with increasing λ values, and the optimal λ was determined by 10-fold cross-validation (Figure 5A). Four feature genes were ultimately identified, including *Wfdc21*, *B430010I23Rik*, *Ces1f*, and *Tst*. Heatmap analysis demonstrated distinct expression patterns of these genes among the SC, SH, and TH groups (Figure 5B). Compared with the SC group, these genes showed altered expression in the SH group and partial restoration following exercise intervention in the TH group.

In scWAT, LASSO regression identified eight feature genes, including *Rasl11a*, *Ccl2*, *Cd68*, *Acaca*, *Zbtb5*, *Clstn3*, *Adissp*, and *Agpat2* (Figure 5C,D). These genes exhibited consistent expression reversal patterns across the three groups. Among them, *Ccl2* showed increased expression in the SH group and reduced expression following exercise intervention. In addition, immune-related genes such as *Cd68* and metabolism-related genes including *Acaca* and *Agpat2* also exhibited exercise-reversed expression patterns.

The feature genes identified by LASSO regression differed between the two adipose depots. In vWAT, representative genes included *Wfdc21 and Ces1f*, whereas scWAT was characterized by genes including *Ccl2*, *Adissp*, and *Agpat2.*

### 3.6. Tissue-Specific Analysis of Skeletal Muscle

To characterize transcriptional changes associated with exercise intervention in SM, differential genes identified in triceps tissue were further analyzed by functional enrichment analysis, pathway enrichment analysis, and feature gene selection.

#### 3.6.1. Functional Enrichment Analysis

**Figure 6:**
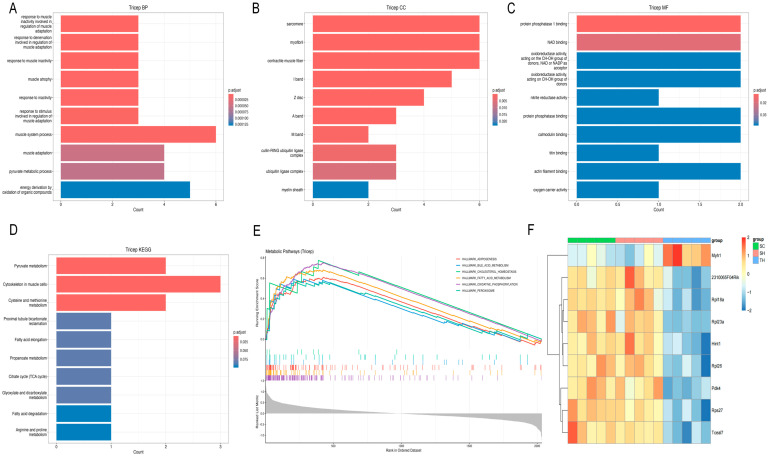
Functional enrichment, immune-related GSEA and core gene expression profiles in SM. (**A**–**D**) GO and KEGG enrichment results including BP (**A**), CC (**B**), MF (**C**) and KEGG pathways (**D**). Bar color corresponds to adjusted *p*-value, and bar length denotes the number of enriched genes. (**E**) GSEA reveals enrichment of inflammatory signaling linked to T cells, B cells, NK cells and monocytes. (**F**) Heatmap of nine LASSO-screened exercise-responsive genes (*Myh1*, *Pdk4*, *Hint1*, *2310065F04Rik*, *Rpl18a*, *Rpl23a*, *Rpl26*, *Rps27*, *Tceal7*). Gene expression is normalized per row by z-score (red = high expression, blue = low expression). The top annotation bar marks experimental groups: SC (green), SH (red), TH (blue).

GO enrichment analysis showed that differential genes in SM were mainly enriched in biological processes including muscle adaptation, muscle atrophy, the muscle system process, and pyruvate metabolic process (Figure 6A). CC terms were primarily associated with muscle structural components, including sarcomere, myofibril, contractile muscle fiber, and Z disc (Figure 6B). MF terms included calmodulin binding, actin filament binding, protein phosphatase binding, and kinase binding (Figure 6C). KEGG pathway analysis demonstrated enrichment in energy metabolism-related pathways, including pyruvate metabolism, glycolysis/gluconeogenesis, the citrate cycle (TCA cycle), and fatty acid elongation (Figure 6D).

#### 3.6.2. GSEA of Metabolic Pathways

GSEA showed that differential genes in SM were enriched in several metabolic pathways, including oxidative phosphorylation, fatty acid metabolism, and adipogenesis (Figure 6E).

#### 3.6.3. LASSO-Based Feature Gene Selection

LASSO regression analysis identified nine feature genes in SM, including *Myh1*, *Pdk4*, *Hint1*, *2310065F04Rik*, *Rpl18a*, *Rpl23a*, *Rpl26*, *Rps27*, and *Tceal7* (Figure 6F). Heatmap analysis showed that these genes exhibited similar expression patterns in the SC and SH groups, whereas distinct expression patterns were observed in the TH group. Among them, *Pdk4*, *Hint1*, *2310065F04Rik*, *Rpl18a*, *Rpl23a*, *Rpl26*, *Rps27*, and *Tceal7* showed lower expression levels in the TH group, while Myh1 showed higher expression levels following exercise intervention.

### 3.7. Cross-Species Validation of Candidate Gene Expression in Human AT

To validate the expression patterns of candidate genes identified in the mouse model, cross-species validation was performed using publicly available human AT datasets.

**Figure 7:**
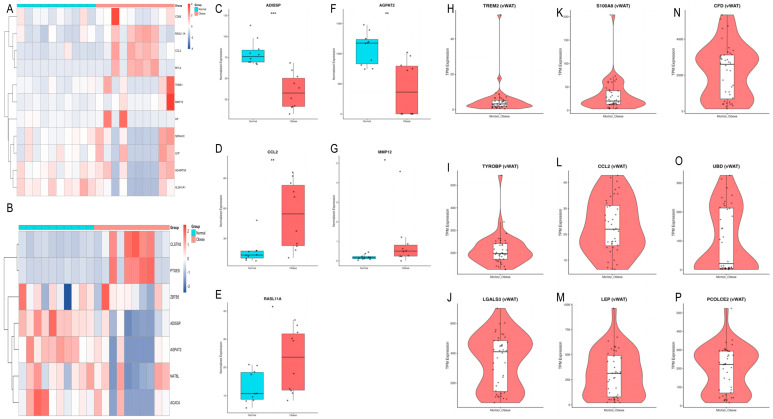
Cross-species validation of candidate gene expression patterns in human scWAT and visceral vWAT. (**A**,**B**) Expression heatmaps of candidate genes in human scWAT comparing normal and obese individuals. (**C**–**G**) Box plots showing differential expression of selected candidate genes (*ADISSP*, *AGPAT2*, *CCL2*, *MMP12*, *RASL11A*) in scWAT between normal and obese groups. (**H**–**P**) Violin plots displaying expression levels of key vWAT candidate genes (*TREM2*, *TYROBP*, *LGALS3*, *S100A8*, *CCL2*, *LEP*, *UBD*, *PCOLCE2*) in morbidly obese individuals. Significance levels: * *p* < 0.05, ** *p* < 0.01, *** *p* < 0.001.

In scWAT, gene expression levels were compared between healthy and obese individuals. *CCL2*, *MMP12*, and *RASL11A* showed significantly higher expression levels in obese individuals, whereas *ADISSP* and *AGPAT2* showed significantly lower expression levels (Figure 7A–G). The expression trends of these genes were consistent with those observed in the mouse model.

In vWAT samples from individuals with morbid obesity, several candidate genes identified in the present study, including *TREM2*, *TYROBP*, *LGALS3*, *S100A8*, *CCL2*, *LEP*, *CFD*, *UBD*, and *PCOLCE2*, showed relatively high expression levels (Figure 7H–P). Among these genes, *CCL2* showed altered expression patterns in both human scWAT and vWAT that were consistent with the mouse data.

## 4. Discussion

In this study, we systematically analyzed transcriptomic changes in multiple tissues under normal, obese, and exercise intervention conditions, with a focus on identifying obesity-induced dysregulated genes that are reversible by exercise. Our results identified a set of exercise-reversed genes in vWAT and scWAT, whereas SM predominantly exhibited adaptive transcriptional changes without clear evidence of expression reversal. These findings indicate that AT and SM respond to exercise stimulation through fundamentally distinct transcriptional regulatory mechanisms [29]. Given that obesity-related pathological alterations contribute to multiple metabolic diseases [30], including insulin resistance, type 2 diabetes, and nonalcoholic fatty liver disease [31], and that exercise is a recognized non-pharmacological intervention for obesity, the tissue-specific regulatory effects and molecular mechanisms of exercise in vWAT, scWAT, and SM remain poorly understood. The following discussion addresses the intra-adipose tissue heterogeneity, the distinct response characteristics of SM, and the cross-tissue comparisons.

Functional enrichment analysis revealed that exercise-reversed genes in AT are primarily involved in immune processes and metabolic regulation. In vWAT, these genes were significantly enriched in chemotaxis, myeloid leukocyte migration, macrophage activation, chemokine signaling, and interleukin-17 signaling pathways. In contrast, scWAT-enriched genes were mainly associated with adaptive thermogenesis, fatty acid metabolism, extracellular matrix remodeling, and AMPK signaling pathways. These results suggest that distinct adipose depots modulate inflammatory status and metabolic homeostasis through depot-specific transcriptional responses to exercise [32]. The pronounced tissue-specific response characteristics between vWAT and scWAT further demonstrate that vWAT-enriched genes are primarily involved in immune-related pathways, whereas scWAT enriches both inflammatory and metabolic pathways, reflecting their distinct physiological functions in inflammation control, energy storage, and metabolic homeostasis maintenance [32,33].

In SM, obesity-associated gene expression dysregulation was not substantially reversed by exercise. Differentially expressed genes were mainly enriched in pathways related to muscle structural organization and energy metabolism, suggesting that SM primarily exhibits adaptive transcriptional responses to exercise [34,35]. Functional enrichment and pathway analyses indicated the involvement of genes in glycolysis, oxidative phosphorylation, and other metabolic pathways, which is consistent with the metabolic functions of SM during exercise adaptation. Collectively, these findings demonstrate that SM and AT possess fundamentally distinct transcriptional response modes to exercise.

A notable observation from our data is that adipose tissues showed clear “reversal” of obesity-associated transcriptional changes following exercise, whereas skeletal muscle exhibited predominantly adaptive transcriptional adjustments without obvious reversal of obesity-related gene expression (largely because few obesity-associated DEGs were detected in muscle in the SH vs. SC comparison at the chosen threshold). A possible interpretation of this distinction is that AT, particularly vWAT, undergoes characteristic pathological changes in obesity—including macrophage infiltration, inflammatory activation, and dysregulated lipolysis—that may be partially corrected by exercise intervention, thereby manifesting as “reversal” of gene expression [36,37,38]. In contrast, SM primarily exhibits functional adaptive decline in obesity (e.g., reduced insulin sensitivity) rather than inflammatory remodeling, and exercise may primarily activate its adaptive programs [39]. This interpretation is further supported by our pathway enrichment results, which showed that obesity-associated dysregulation in AT predominantly involved immune-related pathways (e.g., chemokine and IL-17 signaling), whereas the exercise response in SM was enriched for load-dependent metabolic pathways (e.g., glycolysis and oxidative phosphorylation), consistent with the distinct functional roles of these two tissues. In addition, the apparent lack of reversal in SM may reflect the sensitivity of our analytical approach rather than an absolute biological difference. Furthermore, by our definition, the “exercise-reversed” classification did not require a specific degree of transcriptional recovery toward SC levels, nor did it involve formal statistical testing for obesity–exercise interaction. This intentionally permissive criterion prioritized the identification of directionally corrected candidates, but it also carries a risk of arbitrary classification and does not distinguish complete normalization from partial attenuation. Consequently, the genes identified through this approach should be regarded as candidates for further investigation rather than conclusively established reversal markers.

To validate the reliability of our core findings, we performed cross-species validation using publicly available human AT datasets. Multiple genes showed consistent expression trends between mouse and human samples, with *CCL2* exhibiting a consistent dysregulation pattern across tissues and species [40]. It should be noted that, given the transcriptome-only nature of this study, *CCL2* can only be considered a potential regulatory marker and cannot yet be designated as a direct causative/functional target. Protein–protein interaction network analysis identified several core genes involved in exercise-related transcriptional regulation in AT: *Tyrobp*, *Ctss*, *Adgre1*, and *Ccl2* in vWAT, and *Ccl2*, *Lep*, and *Cd68* in scWAT. Notably, *CCL2* showed highly consistent expression patterns across protein–protein interaction network analysis, LASSO-based feature selection, and cross-species validation, suggesting that it is an evolutionarily conserved potential target responsive to exercise regulation. However, as with all genes identified in this purely transcriptomic study, these findings are correlative; functional experiments are required to determine whether *CCL2* plays a causative role in exercise-mediated immune–metabolic remodeling or merely serves as a surrogate marker of tissue adaptation. Furthermore, LASSO regression identified a set of exercise intervention-related signature genes, including *Wfdc21*, *Ces1f*, and *Tst* in vWAT, and inflammatory markers (*Ccl2*, *Cd68*) together with metabolic genes (*Acaca*, *Adissp*, *Agpat2*) in scWAT. However, these genes represent statistically derived transcriptomic features that have not yet been experimentally validated and should not be directly interpreted as biomarkers.

From a clinical perspective, our findings suggest that CCL2-centered immune–metabolic pathways in adipose tissue may represent a molecular axis responsive to exercise intervention in obesity. However, the present data do not permit delineation of which specific patient subgroups might benefit most from this regulatory mechanism. Whether the observed transcriptional “reversal” patterns are preserved in older adults, individuals with type 2 diabetes, metabolic syndrome, or sarcopenic obesity remains unknown, as these populations were not represented in the source dataset. Moreover, the source dataset was derived from young (6-week-old) male C57BL/6JN mice subjected to a relatively short-term high-fat diet (6 weeks) and a specific voluntary wheel-running protocol (3 weeks). This experimental design does not fully recapitulate the clinical reality of obesity, which most frequently presents in older individuals with long-standing metabolic disturbances and multiple comorbidities. Therefore, it remains unknown whether the identified transcriptional patterns are generalizable to: (i) aged populations or metabolically complicated patients; (ii) other exercise modalities (e.g., forced treadmill running, resistance training, or swimming); (iii) different training volumes or intensities; or (iv) distinct stages of obesity (e.g., early-onset vs. long-standing obesity). Future studies incorporating diverse clinical cohorts with detailed metabolic phenotyping, varied intervention protocols, and aged models will be required to determine the translational generalizability of our findings.

Several limitations of this study should be acknowledged. First, our analyses are based on a single publicly available RNA-seq dataset (GSE183239). While our integrative bioinformatic approach successfully identified candidate exercise-responsive genes, these results remain predictive and correlative rather than causal. The observed mRNA-level “reversal” does not guarantee corresponding changes at the protein level or confirm functional contribution to restoring immune–metabolic homeostasis in vivo. Experimental validation, including protein quantification and functional assays (e.g., gene knockout or pharmacological inhibition), is required to establish causal relationships. Additionally, the application of LASSO regression for feature selection was performed on a relatively small sample size (*n* = 15 per tissue), which carries a risk of overfitting; therefore, the selected feature genes should be interpreted as exploratory statistical features for hypothesis generation rather than definitive biomarkers. Second, the bulk RNA-seq data used in this study represent average expression signals across entire tissues. Given that vWAT, scWAT, and SM are highly heterogeneous tissues composed of multiple cell lineages, our tissue-level analysis cannot distinguish whether exercise-induced transcriptional changes originate from parenchymal cells, infiltrating immune cells, or other stromal populations. Single-cell or single-nucleus RNA sequencing would be necessary to dissect cell-type-specific contributions. Furthermore, the cross-species validation using human GEO datasets is limited by fundamental differences in study design (cross-sectional human cohorts vs. interventional mouse models) and incomplete clinical metadata for some datasets (e.g., GSE294150). These comparisons were intended solely to assess the conservation of expression directionality in obesity, and should be viewed as supportive correlative evidence rather than confirmatory proof of conserved regulatory mechanisms. Third, the present results should be considered hypothesis-generating rather than conclusive, as discussed above regarding the clinical and translational scope. Future studies integrating animal experiments, single-cell technologies, and prospective exercise intervention cohorts are warranted to further validate and extend our conclusions.

## 5. Conclusions

In summary, this study systematically characterized the tissue-specific transcriptomic responses to exercise intervention in obesity using multi-tissue transcriptomic analysis combined with bioinformatics and machine learning approaches. Exercise induced distinct regulatory patterns across adipose depots and SM. In vWAT, exercise was primarily associated with suppression of immune–inflammatory pathways, whereas scWAT exhibited combined regulation of inflammatory responses and metabolic remodeling pathways. In contrast, SM mainly displayed adaptive transcriptional changes related to energy metabolism and structural remodeling.

Through differential expression analysis and LASSO-based feature selection, we identified several candidate exercise-responsive genes. Subsequent functional enrichment, PPI network analysis, and GSEA further characterized their involvement in immune–inflammatory and metabolic pathways. Among the candidates, *CCL2* showed consistent expression patterns across adipose depots and species, highlighting it as a conserved candidate associated with exercise-mediated immune–metabolic remodeling. However, the present findings are hypothesis-generating in nature, and the identified candidates—particularly *CCL2*—require further validation in aged animal models, diverse clinical populations, and prospective exercise intervention studies to confirm their reproducibility and translational relevance. Functional experiments are also warranted to establish causal relationships.

Overall, these findings provide a transcriptomic framework for understanding the tissue-specific molecular responses to exercise in obesity and offer candidate targets for future mechanistic and translational research, while acknowledging that the exploratory scope of this study necessitates independent experimental validation.

## Figures and Tables

**Table 1:** Summary of genes whose obesity-induced dysregulation was reversed by exercise intervention in vWAT and scWAT.

Tissue	Regulatory Pattern	Gene Names	Numbers
vWAT	Obesity-upregulated → exercise-downregulated	*Adgre1*, *Atp1a4*, *C3ar1*, *Ccdc62*, *Ccl2*, *Ccl7*, *Ccl9*, *Cd300c2*, *Cd44*, *Cd52*, *Cd53*, *Cd84*, *Clec4a3*, *Ctss*, *Dpep2*, *Ear2*, *Fcgr3*, *Hp*, *Lctl*, *Lep*, *Lgals3*, *Lgmn*, *Lilrb4a*, *Mpeg1*, *Ms4a7*, *Pcolce2*, *Rab7b*, *Rac2*, *S100a8*, *Saa3*, *Sfrp5*, *Tph2*, *Trem2*, *Tulp3*, *Tyrobp*, *Ubd*, *Wfdc21*	37
	Obesity-downregulated → exercise-upregulated	*B430010I23Rik*, *Ces1f*, *Cfd*, *Cldn22*, *Dennd2d*, *Fam13a*, *Tst*, *Vnn3*	8
scWAT	Obesity-upregulated → exercise-downregulated	*1700047G03Rik*, *Adamts5*, *Aldh1a1*, *Ccl2*, *Ccl7*, *Ccl9*, *Cd68*, *Hp*, *Lep*, *Mmp12*, *Mt2*, *Myl4*, *Rasl11a*, *Sacd3*, *Sema3c*, *Sfrp5*, *Thbs1*, *Ubd*	18
	Obesity-downregulated → exercise-upregulated	*Acaca*, *Adissp*, *Agpat2*, *Clstn3*, *H2-Q10*, *Nat8l*, *Ptges*, *Zbtb5*	8
vWAT + scWAT	Common exercise-reversed targets in both depots	*Ccl2*, *Ccl7*, *Ccl9*, *Hp*, *Lep*, *Saa3*, *Sfrp5*, *Ubd*	8

Genes are grouped by tissue and regulatory pattern: (1) obesity-upregulated genes downregulated by exercise, and (2) obesity-downregulated genes upregulated by exercise. The “common targets” row lists the 8 genes that showed consistent reversal in both depots.

## Data Availability

The datasets analyzed during the current study were downloaded from the public GEO database (GSE183239, GSE162653, GSE294150). All relevant data are available from the corresponding public repository.

## Abbreviations

The following abbreviations are used in this manuscript:

AT	Adipose tissue
EVs	Extracellular vesicles
miRNAs	microRNAs
SM	Skeletal muscle
vWAT	Visceral white adipose tissue
scWAT	Subcutaneous white adipose tissue
GEO	Gene Expression Omnibus
SC	Normal diet control
SH	High-fat diet-induced obesity
TH	Aerobic exercise intervention
GO	Gene Ontology
BP	Biological process
CC	Cellular component
MF	Molecular function
KEGG	Kyoto Encyclopedia of Genes and Genomes
PPI	Protein–protein interaction
LASSO	Least absolute shrinkage and selection operator
GSEA	Gene set enrichment analysis
NES	Normalized enrichment score
FDR	False discovery rate
DEGs	Differentially expressed genes
